# Gene expression profile and cancer-associated pathways linked to progesterone receptor isoform a (PRA) predominance in transgenic mouse mammary glands

**DOI:** 10.1186/s12885-018-4550-z

**Published:** 2018-06-25

**Authors:** María José Carlini, María Sol Recouvreux, Marina Simian, Maria Aparecida Nagai

**Affiliations:** 1 Discipline of Oncology, Department of Radiology and Oncology, Faculty of Medicine, University of São Paulo, São Paulo, SP 01246-903 Brazil; 2 Laboratory of Molecular Genetics, Center for Translational Research in Oncology, Cancer Institute of São Paulo, São Paulo, SP 01246-000 Brazil; 3 Instituto de Oncología “Ángel H. Roffo”, Av. San Martín 5481, C1417DTB Ciudad Autónoma de Buenos Aires, Argentina; 4 Present address: Department of Pharmacological Sciences, Icahn School of Medicine at Mount Sinai, 1468 Madison Avenue, New York, NY 10029 USA; 5 Present address: Instituto de Nanosistemas, Universidad Nacional de San Martín, Av. 25 de Mayo 1021, 1650 San Martín, Provincia de Buenos Aires Argentina

**Keywords:** Progesterone receptor, Isoforms, Transgenic mice, Mammary gland, Hyperplasia, Breast cancer, Gene expression profiling, Gene set enrichment analysis, Biomarker

## Abstract

**Background:**

Progesterone receptor (PR) is expressed from a single gene as two isoforms, PRA and PRB. In normal breast human tissue, PRA and PRB are expressed in equimolar ratios, but isoform ratio is altered during malignant progression, usually leading to high PRA:PRB ratios. We took advantage of a transgenic mouse model where PRA isoform is predominant (PRA transgenics) and identified the key transcriptional events and associated pathways underlying the preneoplastic phenotype in mammary glands of PRA transgenics as compared with normal wild-type littermates.

**Methods:**

The transcriptomic profiles of PRA transgenics and wild-type mammary glands were generated using microarray technology. We identified differentially expressed genes and analyzed clustering, gene ontology (GO), gene set enrichment analysis (GSEA), and pathway profiles. We also performed comparisons with publicly available gene expression data sets of human breast cancer.

**Results:**

We identified a large number of differentially expressed genes which were mainly associated with metabolic pathways for the PRA transgenics phenotype while inflammation- related pathways were negatively correlated. Further, we determined a significant overlap of the pathways characterizing PRA transgenics and those in breast cancer subtypes Luminal A and Luminal B and identified novel putative biomarkers, such as PDHB and LAMB3.

**Conclusion:**

The transcriptional targets identified in this study should facilitate the formulation or refinement of useful molecular descriptors for diagnosis, prognosis, and therapy of breast cancer.

**Electronic supplementary material:**

The online version of this article (10.1186/s12885-018-4550-z) contains supplementary material, which is available to authorized users.

## Background

Estrogen and progesterone signaling via their receptors play important roles not only in normal mammary gland development but also in breast cancer progression [1, 2]. Gene expression profiling has revealed at least five subtypes of breast cancer: luminal A (LumA), luminal B (LumB), HER2, basal and normal [3, 4]. Importantly, the immunophenotypic evaluation, i.e.*,* the analysis of markers by immunohistochemistry such as estrogen receptor (ER), PR, Ki67, HER2 and basal cytokeratins (CK 5/6, CK14) is a useful surrogate of the gene expression defined-subtypes in the clinical setting [5].

PR is expressed from a single gene as two isoforms, PRA and PRB. The two isoforms are expressed at similar levels in the breast, but the ratio can be altered in human breast tumors, with the PRA isoform predominating [6]. Moreover, high PRA/PRB ratios predicted shorter disease-free survival in patients who received local therapy followed by adjuvant tamoxifen, indicating resistance to tamoxifen and underscoring the prognostic value of discriminating PR isoforms [7, 8]. Importantly, preclinical studies with murine and human tumors and ex vivo human breast cancer tissue culture assays showed that antiprogestin responsiveness in breast cancer is determined by the PRA/PRB expression ratio, specifically, an inhibitory effect of the antiprogestin mifepristone is only obtained in tumors with higher levels of PRA than PRB [9, 10].

In vitro, inducing a high PRA/PRB ratio in the T47D cell line conferred responsiveness to progestins to a set of genes involved in cellular metabolism and regulation of cell shape and adhesion. In accordance, progestin treatment resulted in reduced cell adhesion, which was significantly decreased even further when PRA was predominant [11]. Other studies aiming to determine the relative contributions of PR isoforms functions of PRA and PRB have employed cell lines engineered to express only a single PR isoform [12]. Due to common features, the mouse mammary gland is a useful model for normal human breast, and breast cancer [1] and transgenic mice allow exploring hormone receptor actions in the gland. Transgenic mice carrying either an additional A form of PR (PRA transgenics) or the B form of PR show abnormal mammary gland features [13]. In particular, mammary glands of PRA transgenics exhibit extensive lateral branching, ductal hyperplasia, a disorganized basement membrane and loss of cell-cell adhesion [13–15]. Studies using the molecular markers for transformation, as defined by Medina [16], revealed that these mammary glands contained at least two distinct populations of transformed epithelial cells. The ducts with normal histology contained cells resembling immortalized cells, while hyperplasias consisted of cells in later stages of transformation associated with early pre-neoplasias and exhibited increased epithelial cell proliferation [15]. Similarly, loss of coordinate expression of PRA and PRB occurs early in human breast cancer progression [17]. Therefore, evidence supports that misregulation of the PRA/PRB expression ratio can have major implications for mammary carcinogenesis.

In the present study, using the well characterized PRA transgenic mouse model, we sought to determine the full repertoire of target transcripts and pathways underlying the aberrant phenotype of mammary glands in PRA transgenics as compared with wild-type litter-mates in a relevant in vivo microenvironment under physiological hormone conditions. Further, using publicly available gene expression data sets of human breast cancer, we have explored the potential overlapping relevant pathways with breast cancer and identified novel putative biomarkers. Understanding the molecular context of deregulated PR action in the mammary gland may well accelerate the formulation of useful molecular descriptors for diagnosis, prognosis, and therapy of breast cancer.

## Methods

### Mice

Nulliparous adult FVB mice (20–25 weeks) were used in this study. The generation of PR-A transgenic mice, which carry an imbalance in the normal ratio of the two forms of PR by overexpression of the A form, has been previously described [13]. In brief, we used a binary transgenic system in which the GAL-4 gene, driven by the murine cytomegalovirus (CMV) promoter (CMV-GAL-4 mice), served as the transactivator of the PR-A gene, carrying four GAL-4-binding sites (UAS; UAS-PR-A mice). Crossing the CMV-GAL-4 mice with UAS-PR-A mice resulted in bigenic mice carrying additional PR-A gene [13]. The animals were housed in the Animal Care Division at the Institute of Oncology “Ángel H. Roffo” in an air-conditioned room at 22 °C under a 12-h light/dark cycle with access to food and tap water ad libitum and treated in accordance with the NIH Guide for Humane Use of Animals in Research.

### Harvesting mammary gland RNA

Mice were euthanized by cervical dislocation, and mammary glands were harvested from all mice at diestrus, given that serum progesterone levels and the morphological grade, epithelial proliferation, and apoptosis in the mammary gland all peak at this stage [18]. A total of seven animals were used (3 wild-type, 4 PRA transgenic). In each case, a single abdominal gland was removed following excision of lymph nodes and immediately frozen in liquid nitrogen. Tissue was pulverized in a Thermo-vac tissue pulverizer (Thermovac Industries Corp.) at liquid nitrogen temperature. The resulting powder was transferred into tubes containing 1.5 ml of Trizol reagent (Thermo Fisher Scientific Inc.) and homogenized by passing the lysate through sterile, disposable 21G needle 5 times twice. Following homogenization, samples were centrifuged at 12000×g for 10 min at 4 °C. The fatty layer above the supernatant was removed and discarded, and the cleared supernatant was transferred to a new tube. Total RNA was extracted according to manufacturer’s instructions, followed by additional column purification (RNeasy Mini Kit, Qiagen Inc.). We chose a reference-based 2-color microarray design given that it decreases intra- and inter-experimental variability by relating expression measurements of experimental RNA to a common reference, rather than relying on absolute signal intensity. At the same time, the use of spike-in controls allowed to monitor the system for linearity, sensitivity, and accuracy (https://www.genomics.agilent.com/files/Manual/Spike-in_Kit.pdf). RNA integrity was assessed using the RNA 6000 Nano Assay and Agilent 2100 Bioanalyzer (Agilent Technologies Inc.).

### Microarray analysis

Labeled cRNA was prepared from 50 ng RNA using the Low Input QuickAmp Labeling Kit Two-Color and RNA Spike-In Kit for Two colors v4.0 (Agilent Technologies Inc.) according to manufacturer’s instructions, followed by RNeasy Mini Kit (Qiagen Inc.) purification. Experimental samples were labeled with Cy5 and Universal Mouse Reference RNA (Agilent Technologies Inc.) with Cy3. Dye incorporation and cRNA yield were checked with the NanoDrop Spectrophotometer (NanoDrop Technologies Inc.). Then, 300 ng of Cy5/Cy3-labeled cRNA was fragmented and prepared for hybridization using the Gene Expression Hybridization Kit (Agilent Technologies Inc.) following manufacturer’s instructions and hybridized to SurePrint G3 Mouse Gene Expression v2 8x60K (Agilent Technologies Inc.) for 17 h at 65 °C in a rotating hybridization oven. After hybridization, microarrays were washed for 1 min at room temperature with GE Wash Buffer 1 (Agilent Technologies Inc.) and 1 min with 37 °C GE Wash buffer 2 (Agilent Technologies Inc.) containing 0.005% Triton X-100. Slides were scanned immediately after washing using the G4900DA SureScan microarray scanner system, features were extracted with Agilent Feature Extraction Software (Agilent Technologies Inc.) and good quality control metrics were confirmed for all report files. Data analyses were conducted with GeneSpring GX software (Agilent Technologies Inc.). Input data was pre-processed by baseline transformation to the median of all samples. After grouping of replicates according to their respective experimental condition differential gene expression was statistically determined by unpaired T-test with significance set at *p* < 0.05. An unbiased grouping of samples was created only on the basis of their molecular profiles by unsupervised clustering analysis. Transcripts were subjected to hierarchical clustering with Euclidean distance metrics and average linkage using GeneSpring. In the supervised analysis, the transcripts identified as differentially expressed were used for hierarchical clustering as described above.

### Gene ontology analysis

Statistically overrepresented GO categories within differentially expressed gene lists were determined using BiNGO (Biological Network GO) [19] and visualized using Cytoscape. The statistical test was the Hypergeometric test with Benjamini & Hochberg False Discovery Rate (FDR) correction and 0.05 significance level. GO annotation and ontology files were from the GO consortium (www.geneontology.org), and the reference set included all the genes on the microarray.

### Gene set enrichment analysis

GSEA [20] was carried out by using the GSEA software, version 3.0, obtained from the Broad Institute (http://www.broadinstitute.org/gsea/downloads.jsp). Expression data sets (.GCT format), phenotype labels (.CLS format) and annotations (.CHIP format) were created according to GSEA specifications. We computed overlaps with the H (hallmark gene sets), C2 (curated gene sets) and C5 (GO gene sets) collections. The C2 collection analysis was divided for the subcollections cp (canonical pathways: Biocarta, KEGG, and Reactome) and CGP (chemical and genomic perturbations), the latter was filtered by the search “mammary OR breast” and “*Mus musculus*” and “*Homo sapiens*” organisms, resulting in a collection of 442 gene sets. Gene set permutations (to avoid the potential problem of a small sample size) were done 1000 times for each analysis using the weighted enrichment statistic and signal to noise metric. Gene sets that met the false discovery rate lower than 25% criterion were considered significant. When necessary, enrichment maps [21] were plotted to visualize GSEA analysis results and overcome gene set redundancy.

For comparison purposes, GSEA files were prepared for breast cancer gene expression data (METABRIC, [22, 23], downloaded from cBIO portal [24, 25] using the PR+ samples in LumA and LumB subtypes, and the Basal and Her2+ subtypes compared with Normal. GSEA was computed with the H collection. The pathway list overlap between the significant pathways in PRA transgenics and the breast cancer subtypes was calculated with overlap stats program (http://www.nemates.org/MA/progs/overlap_stats.html) which calculates the significance of the overlap (*P* value) by using hypergeometric probability.

### Kaplan-Meier curves

Kaplan-Meier plotter (http://kmplot.com/analysis/) was used to perform a meta-analysis based biomarker assessment. Relapse-free survival curves were generated with patients split according to the best cutoff values auto-selected by the tool, using only JetSet best probe sets, removing redundant probes and excluding biased arrays. *P* values are from log-rank test.

## Results

### Transcriptional changes in PRA transgenic mammary glands

**Fig. 1:**
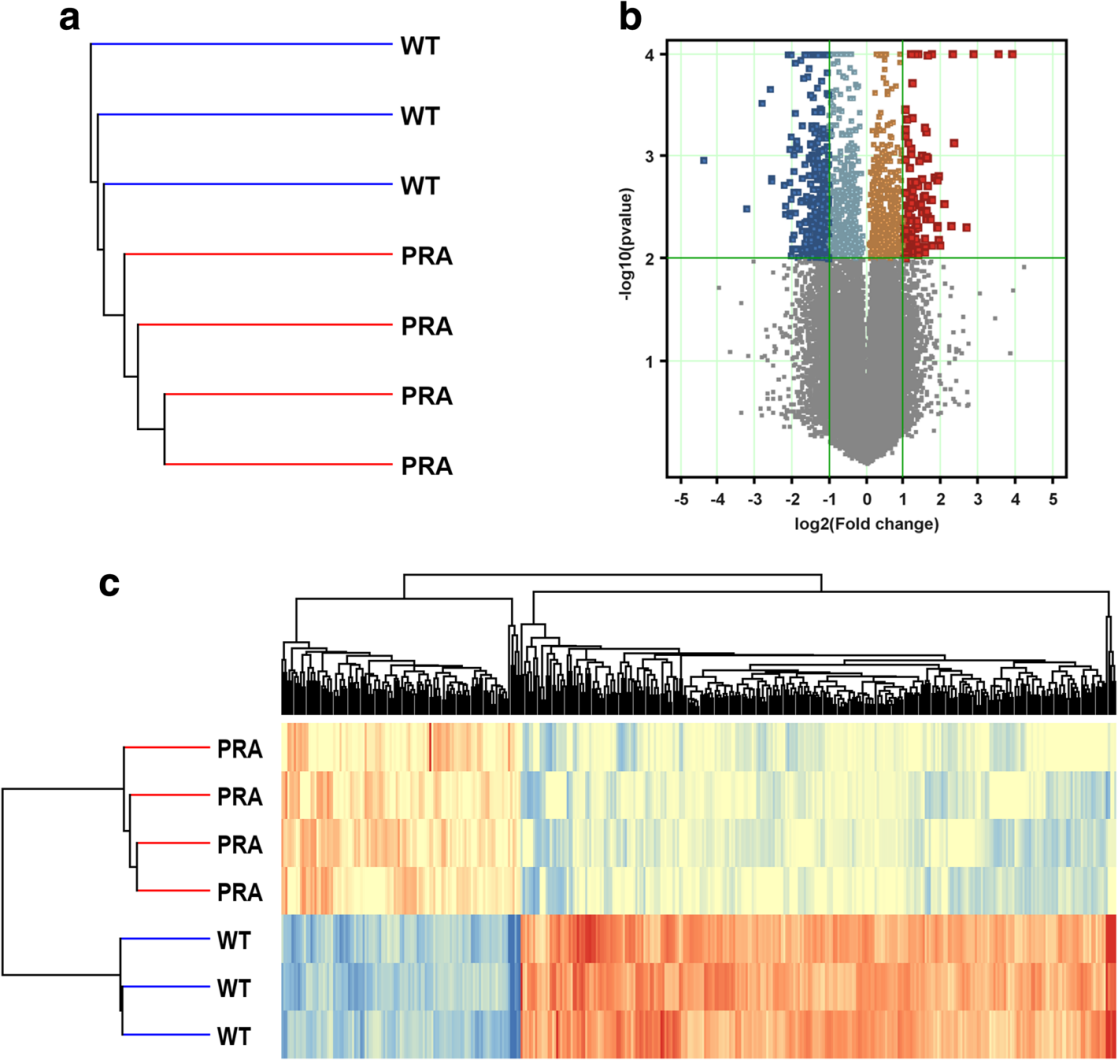
**a**) Dendrogram depicting unsupervised clustering of array data for 4 PR-A transgenics and 3 wild-type samples. **b**) Volcano plot for differentially expressed genes (DEG). DEG appear above the green horizontal line (unpaired t-test, *P* < 0.01). Genes induced > 2-fold are on the right of the right green vertical line (red colored), and the ones repressed > 2-fold are on the left of the left green vertical line (blue colored). **c**) Heat map depicting the relative fold change in expression levels of the 401 transcripts that were differentially expressed by ≥2.0-fold (*P* < 0.01, 255 downregulated, blue and 92 upregulated, red) between the PRA transgenic and the wild-type group

To better understand the effects of PRA overexpression on breast cancer, we compared the expression profile in mammary glands of PRA transgenics with wild-type littermates using oligonucleotide microarray analysis. The expression signatures for wild-type and PRA transgenic mammary glands were strong enough to be recognized by unsupervised clustering (Fig. 1a). A volcano plot representing the distribution of the fold changes (FC) and *P*-values, showed that 401 of 56,344 probes were significantly (*P* < 0.01, unpaired t-test, FC ≥ 2) different in PRA transgenic mice as compared with wild-type (255 down and 92 up-regulated, Fig. 1b). Focusing on these most differentially expressed genes (DEG), we applied clustering across samples and genes, using the Euclidean distance metric and average-linkage and plotted a heat map, where the genes (columns) and samples (rows) are ordered by their corresponding hierarchical clusters (Fig. 1c). As expected, we observed robust gene clusters from replicate data sets.

### Gene ontology analysis

**Fig. 2:**
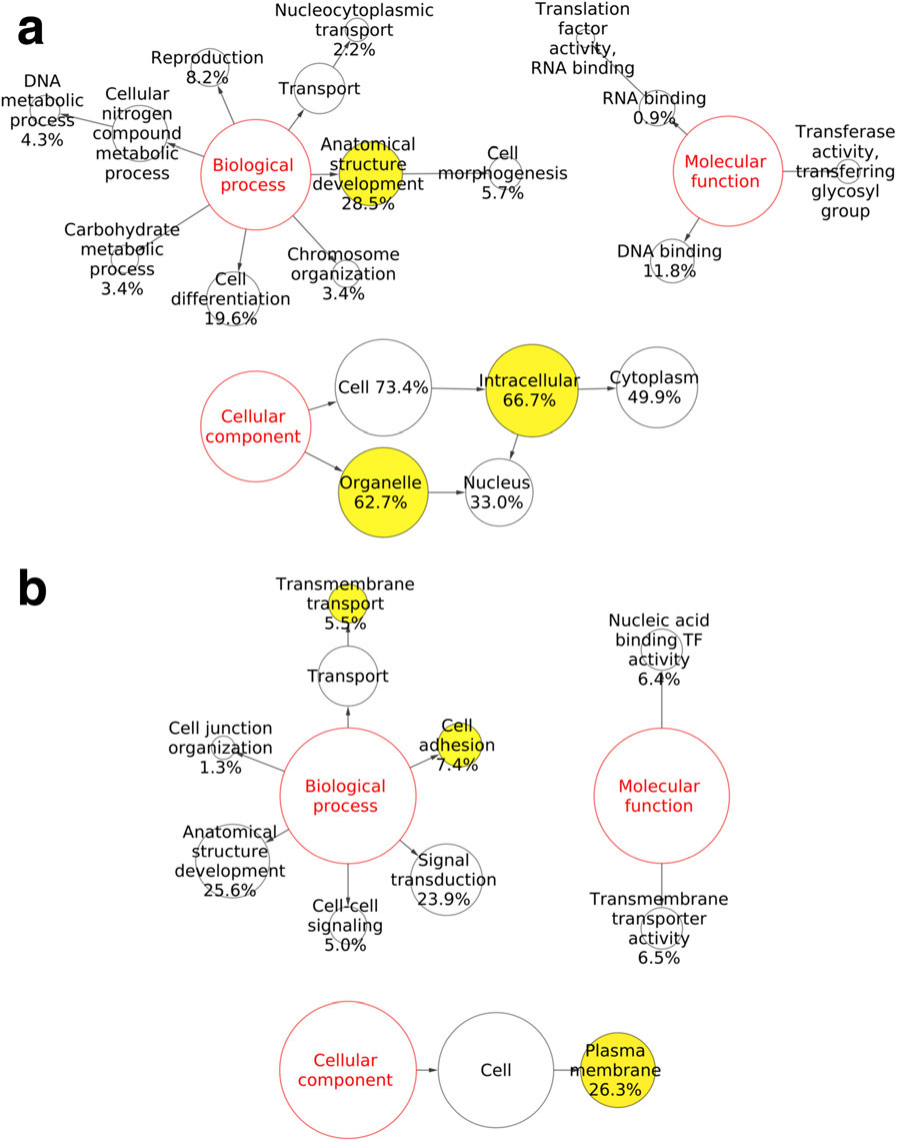
BiNGO results visualized as Cytoscape graphs for **a**) down-regulated genes and **b**) up-regulated genes in PRA transgenic mammary glands. Yellow nodes represent GO categories that are overrepresented at the significance level (*P* < 0.05, Hypergeometric Test with Benjamini & Hochberg’s False Discovery Rate correction). The size of the node is related to the number of genes in the cluster belonging to a certain GO category

DEGs were interrogated for their GO classes using BiNGO (Biological Network GO) to identify overrepresented functional themes, using the complete list of transcripts in the microarray as a reference set. We could not identify significantly enriched categories with this first approach. Therefore we used a less stringent cut-off (≥1.5 FC and *P* < 0.05) to define the DEG list. BiNGO mapping outputs are presented as Cytoscape graphs (Fig. 2). For the upregulated genes, the anatomical structure development category amongst the biological processes was significantly overrepresented (corrected *P* < 0.05), suggesting an active remodeling process (Fig. 2a). As for downregulated genes (Fig. 2b), there were changes mainly at the plasma membrane, as indicated by over-representation of this cellular compartment (corrected *P* < 0.01). Consequently, the enriched cellular processes included transmembrane transport and cell adhesion (corrected *P* < 0.001).

Also, as an in silico approach to validate the microarray data generated in the present study, we compared our DEG data set with PR regulated genes identified in a previous study. Fifty-five genes of our list (≥1.5 FC and *P* < 0.05) had been previously identified as regulated by PR in a human breast cancer cell line model that allows controlled expression of PRA/PRB [26] (Table 1).

**Table 1:** Differentially expressed genes in our gene set supported by previous data [26]

Upregulated (12)	Downregulated (43)
CACNG7 DOCK4 FAM46A GLIS3 KDM4A NEBL NLRP3 ODC1 PITX2 STEAP2 TLR4 TOM1L2	ATP8B1 BTNL2 CAV3 CDC14A DDIT4 ETS1 FAM84A FNDC5 GIGYF2 GPR39 HDAC9 IER3 IL7R KLF2 KLRC1 LAMB3 LIMCH1 LYPD1 MALT1 MAMLD1 NAV2 NEK10 NR4A1 PCDH17 PDE1C PDE4B PDE4DIP PDK4 PDZD2 RGS7 ROR1 SDK1 SH3TC2 SHISA3 SLC16A9 SNCAIP SOCS1 SYT12 TACC2 TNS1 TSGA10 TTC39A VAMP1 ZFYVE28

### Gene set enrichment analysis

GSEA was performed for the PRA > wild-type comparison using C2 (curated gene sets), C5 (GO gene sets) and the H (hallmark gene sets) collections in MSigDB. We chose to interrogate the C2 collection to identify relevant pathways, C5 to confirm and extend our previous GO analysis and H collection for detecting specific biological processes, given that the latter was developed by a combination of automated approaches and expert curation and is more effective and accurate by reducing noise and redundancy [27].

**Fig. 3:**
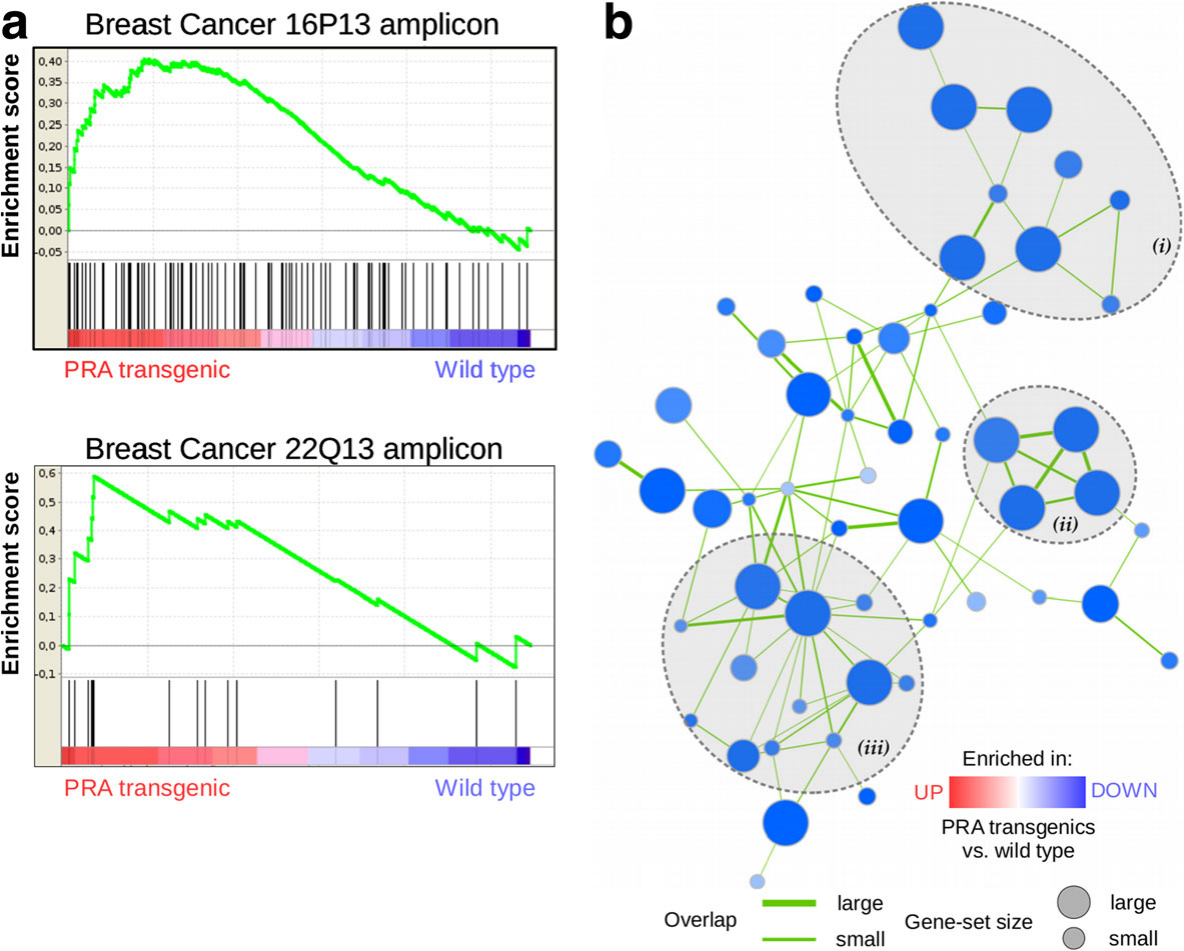
**a**) Enrichment plots showing the correlation of gene sets from breast cancer amplicons 16p13 (FDR q-value 0.106) and 22q13 (FDR q-value 0.101) with the PRA phenotype. Profile of the running enrichment score and positions of gene set members on the rank-ordered list (upregulation or downregulation of genes in PRA transgenics relative to their expression in wild-type). **b**) Enrichment map visualization of gene-sets correlating with down regulated genes in PRA transgenics showing manually identified clusters defined as (i) gene sets related to mammary gland morphological changes or breast cancer subtypes; (ii) gene sets related to estradiol or tamoxifen response and (iii) gene sets comprising targets of polycomb complexes. In the similarity network node size represents the number of genes in the gene-set; edge thickness is proportional to the overlap between gene-sets and the enrichment score is mapped to the node color as a color gradient, in this case blue (high enrichment in wild-type)

The C2 collection (chemical and genomic perturbation -CGP- sub-collection) was limited to the specific context of the mammary gland, employing a search with the terms “mammary OR breast” in the MSigDB resource. Three gene sets were positively correlated with the PRA phenotype, including two sets of genes within amplicons 16p13 and 22q13 identified in a study of 191 breast tumor samples [28]; the corresponding enrichment plots are depicted in Fig. 3a. Of note, amplicon 16p13 is one of the most frequent and well-characterized amplicons in human breast cancer. A high number of gene sets (115) were correlated with the wild-type phenotype. To overcome gene-set redundancy and help in the interpretation we used enrichment map visualization and three approximately homogeneous clusters were manually identified as: (i) gene sets related to mammary gland morphological changes or breast cancer subtypes; (ii) gene sets related to estradiol or tamoxifen response and (iii) gene sets comprising targets of polycomb complexes (Fig. 3b).

Finally, analysis of the H collection revealed eight pathways positively correlated with the PRA phenotype, including metabolism-related pathways (cholesterol homeostasis, metabolism of fatty acids, oxidative phosphorylation, glycolysis and mTORC1 signaling), as well as genes important for mitotic spindle assembly and a subgroup of genes regulated by MYC-version 2 (v2). Nineteen gene sets were positively correlated with the wild-type phenotype (downregulated in PRA). Four gene sets comprised genes involved in inflammation-related pathways. The remaining included hallmark pathways of apoptosis and UV response, epithelial-to-mesenchymal transition (EMT) and TGF-beta signaling, and the developmental hedgehog signaling pathway. The complete list of pathways is presented in Table 2.

**Table 2:** Significant up or downregulated pathways of the hallmark MSigDB collection in PRA transgenic mammary glands, according to GSEA analysis with FDR < 25%

	Pathways	NES	NOM p-val	FDR q-val
Upregulated	OXIDATIVE PHOSPHORYLATION	1.90	0.00	0.003
	ADIPOGENESIS	1.40	0.01	0.139
	MITOTIC SPINDLE	1.40	0.01	0.124
	GLYCOLYSIS	1.30	0.05	0.219
	CHOLESTEROL HOMEOSTASIS	1.30	0.12	0.224
	DNA REPAIR	1.20	0.10	0.259
	MTORC1 SIGNALING	1.20	0.08	0.234
	MYC TARGETS V2	1.20	0.17	0.231
	FATTY ACID METABOLISM	1.20	0.15	0.221
Downregulated	TNFA SIGNALING VIA NFKB	−2.20	0.00	0.000
	IL6 JAK STAT3 SIGNALING	−2.00	0.00	0.001
	ALLOGRAFT REJECTION	−1.90	0.00	0.003
	INFLAMMATORY RESPONSE	−1.80	0.00	0.003
	TGF BETA SIGNALING	−1.70	0.00	0.005
	KRAS SIGNALING UP	−1.60	0.00	0.025
	APOPTOSIS	−1.60	0.00	0.025
	EPITHELIAL MESENCHYMAL TRANSITION	−1.60	0.00	0.023
	UV RESPONSE DN	−1.60	0.00	0.020
	HEDGEHOG SIGNALING	−1.60	0.04	0.025
	INTERFERON GAMMA RESPONSE	−1.50	0.01	0.034
	IL2 STAT5 SIGNALING	−1.40	0.02	0.100
	COMPLEMENT	−1.30	0.04	0.125
	KRAS SIGNALING DOWN	−1.30	0.07	0.212
	HYPOXIA	−1.30	0.06	0.205
	APICAL SURFACE	−1.30	0.18	0.214
	APICAL JUNCTION	−1.30	0.07	0.203
	PI3K AKT MTOR SIGNALING	−1.20	0.13	0.239
	G2M CHECKPOINT	−1.20	0.11	0.248

*NES* normalized enrichment score, *NOM p-val* nominal *p*-value, *FDR q-val* false discovery rate q-value

The results of querying the C2.CP Reactome and KEGG pathways subcollections reinforced the findings obtained with the Hallmark collection. Interestingly, the steroid hormone biosynthesis KEGG pathway correlated with PRA downregulated genes (FDR = 0.106). Also, as expected, C5 GO collection provided similar results as BinGO, thus cross-checking our first analysis.

### Overlapping hallmark pathways in human breast cancer subtypes

It has been reported that the distinct stages of human breast cancer progression premalignant, preinvasive and invasive have remarkable similar transcriptomes with the most significant gene expression changes taking place in the early stages [29, 30]. Porter et al. reported that the most dramatic changes occurred at the normal to ductal carcinoma in situ (DCIS) transition, including the uniform downregulation of 34 genes; while no “in situ” or “invasive” signature was clear [30]. Further, aberrant PR isoform ratios are detected early in the progression of breast lesions from the normal state to malignancy [17]. In view of this, we aimed to assess to what extent the key pathways described above for the PRA > wild-type comparison (Table 2) were similarly enriched in human breast cancer tumor samples of different subtypes. To this end, we determined the enriched pathways for breast cancer subtypes using the transcriptomic data from a much larger data set (METABRIC, [22, 23]) downloaded from cBioPortal [24, 25]. We run GSEA analysis using the hallmark collection with this data, comparing the subtypes mainly positive for PR, LumA and LumB, with normal. Then, we calculated the overlap of the obtained pathways with those identified in our study (Table 3). Of note, we observed a significant overlap of upregulated pathways between the mammary glands of PR-A transgenic and the human LumB breast cancer subtype. Further, the downregulated pathways overlapped significantly with both LumA and LumB subtypes, while no overlapping was observed with the pathways associated with the non-PR expressing subtypes Her2 and Basal (data not shown). To narrow down further analysis we focused on the most upregulated and downregulated pathways: oxidative phosphorylation and TNFA signaling via NFKB, respectively to study the participating genes in more detail.

**Table 3:** Significantly up and downregulated hallmark pathways for breast cancer subtypes and statistical significance of the overlap with those found for PRA transgenics

		Number of significant pathways in the set (out of 50 total)	Number of overlapping pathways	*P* value
Upregulated	PRA Transgenic	8		
	Luminal A	10	3	0.188
	Luminal B	15	5	**0.043**
Downregulated	PRA Transgenic	19		
	Luminal A	28	16	**0.002**
	Luminal B	24	15	**7.10E-04**

Bolded values indicate statistically significant overlap (i.e., *p*-value < 0.05)

### Genes from the oxidative phosphorylation pathway

**Fig. 4:**
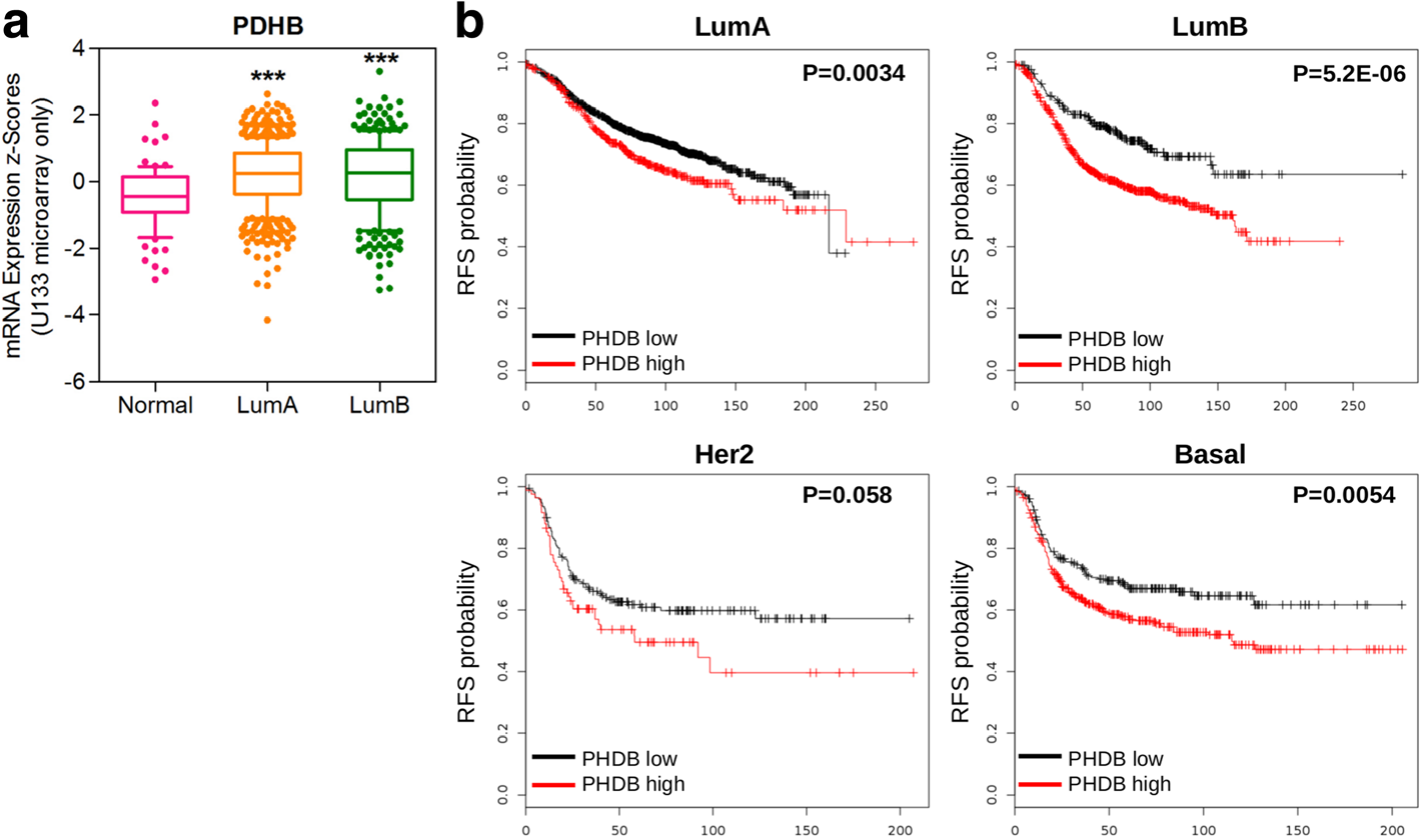
**a**) PDHB expression, from the oxidative phosphorylation pathway, in samples from the METABRIC study, classified by subtype. ***P < 0.001 One-way ANOVA Tukey’s multiple comparison test. **b**) Relapse-free survival curves according to PDHB expression for the different molecular subtypes

**Fig. 5:**
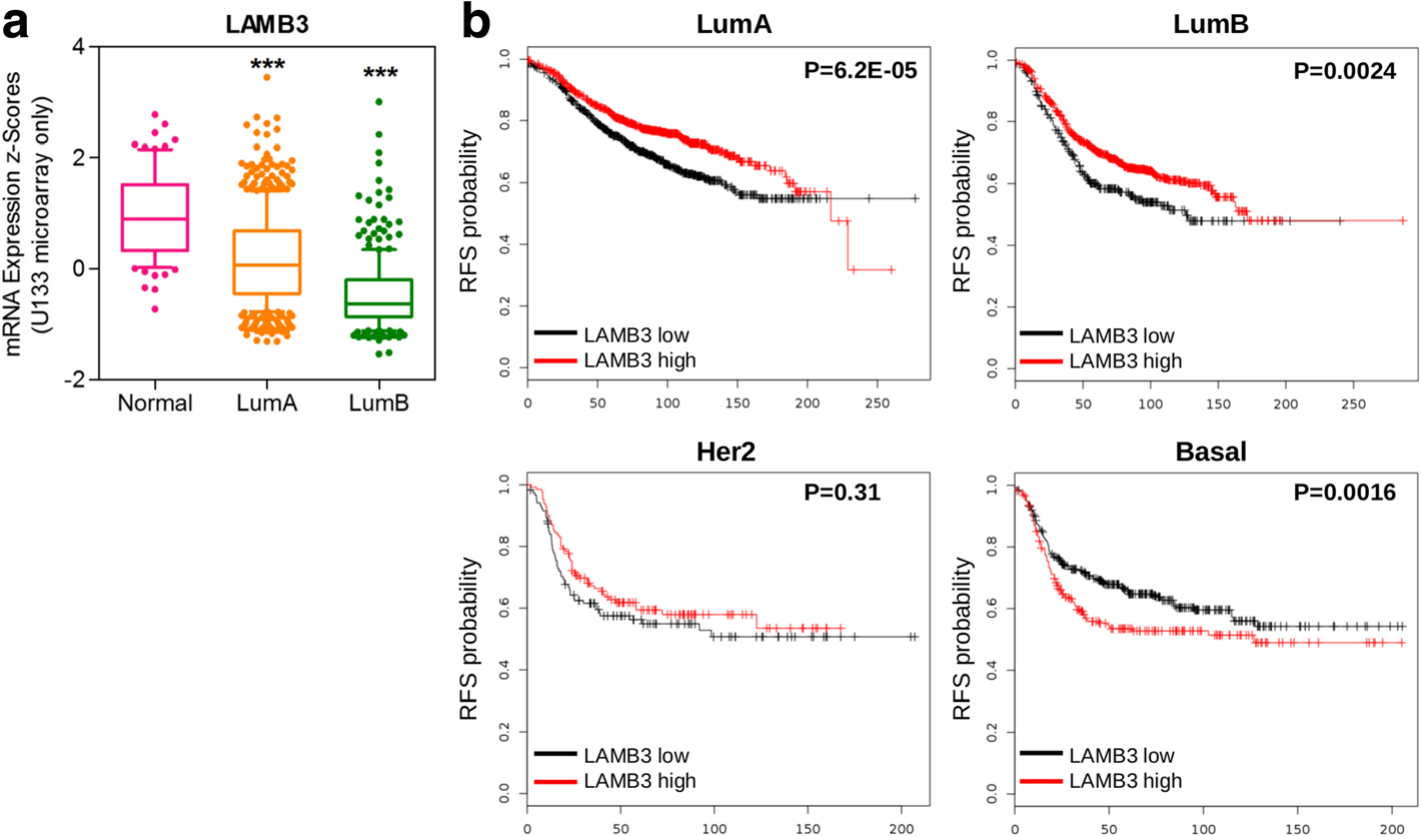
**a**) LAMB3 expression, from the TNFA signaling through NFKB pathway, in samples from the METABRIC study, classified by subtype. ***P < 0.001 One-way ANOVA Tukey’s multiple comparison test. **b**) Relapse-free survival curves according to LAMB3 expression for the different molecular subtypes

When comparing genes obtained by GSEA statistic computation and Genespring (which we used to obtain our list of DEG) we found only two genes from the oxidative phosphorylation pathway overlapping between PRA transgenics and LumB subtype that were in both lists (Table 4 and Additional file 1: Table S1 for a complete list of the genes identified by GSEA analysis). However, one of these (TIMM9) was not significantly modulated in the breast cancer dataset, neither had an impact in patient survival (Additional file 1: Figure S1). On the other hand, PDHB (Pyruvate dehydrogenase E1 component subunit beta, mitochondrial) upregulated in PRA transgenics (FC 1.43, *P* = 0.02) although with a FC at the borderline of our criteria (≥1.5 FC and *P* < 0.05) was not only upregulated in LumB subtype from the METABRIC study, as expected from GSEA analysis, but also in LumA subtype (Fig. 5a). Moreover, we explored the potential prognostic value of PDHB using Kaplan Meier plotter [31] and found that high PDHB expression correlated with poor relapse-free survival for patients with LumA, LumB and Basal tumor subtypes (Fig. 4b).

**Table 4:** Genes contributing to the upregulated oxidative phosphorylation pathway and the downregulated TNFA signaling through NFKB pathway in PRA transgenics and LumA/LumB subtypes

Gene symbol	FC	*P*-value
TIMM9	1.8	0.019
PDHB	1.4	0.024
IL7R	−4.6	0.036
KYNU	−3.1	0.006
EGR3	−2.9	0.029
EGR1	−2.9	0.037
MSC	−2.4	0.022
LAMB3	−2.3	0.001
BMP2	−2.0	0.019
PDE4B	−2.0	0.018
NR4A1	−1.9	0.032
FOSB	−1.9	0.011
KLF2	−1.8	0.041
PMEPA1	−1.8	0.043
JUNB	−1.6	0.009
CD44	−1.6	0.035
CCL2	−1.5	0.001
IER2	−1.5	0.047
ZFP36	−1.5	0.029

*P*-value and fold change (FC) of PRA/wild-type comparison

### Genes from the TNFA signaling via NFKB pathway

For this pathway, we found several genes from our original list overlapping with the LumA and LumB phenotypes (Table 4 and Additional file 1: Table S2 for a complete list of the genes identified by GSEA analysis). With the only exception of CD44, when analyzing the expression data for these genes in the METABRIC study, they were downregulated in the LumA and/or LumB phenotypes, compared with normal as it was expected (9/17 downregulated in both subtypes, 1/17 only in LumA, 6/17 only in LumB). Fig. 5a shows the plot for LAMB3 as an example (see Additional file 1: Figure S2 for the remaining genes). The potential prognostic value of these genes was also assessed, showing promising results for many of them. The Kaplan Meier plots for LAMB3, indicating that low expression was correlated with poor relapse-free survival for patients with LumA and LumB tumor subtypes is shown in Fig. 5b. Globally, low expression of 16/17 genes correlated with poor relapse-free survival in luminal subtype breast cancer (11/17 with both subtypes, 2/17 only with LumB, 3/17 only with LumA, 1 discordant, Additional file 1: Figure S3).

## Discussion

Changes in the native ratio of A to B isoforms of PR have major implications to normal mammary gland biology and also tumorigenesis. For this reason, several groups have previously used expression profiling to identify genes associated with PRA:PRB imbalanced ratio in breast cancer cell lines [11, 26, 32, 33]. However, none have explored the transcriptional changes of preneoplastic lesions that are associated with PRA:PRB imbalance and tumor progression in an in vivo model. In this study, we took advantage of PRA transgenics, a previously described transgenic mouse model where PRA isoform is predominant. Using oligonucleotide microarray technology, we identified the complete repertoire of genes that are altered in expression in the abnormal mammary glands of PRA transgenics and characterized the associated pathways. Importantly, several of the DEG identified in this study (Table 1) had been previously reported as PR targets [26].

Based on the distinct transcriptomics of PRA transgenics, we first identified by GO analysis an enrichment in the biological processes anatomical structure development and cell adhesion for upregulated and downregulated genes, respectively (Fig. 2a and b). This is in accordance with the key morphological features of PRA transgenic mammary glands, including extensive lateral branching, the disruption in the organization of the basement membrane and a decrease in cell–cell adhesion [13]. The influence of PRA:PRB ratio in cell adhesion has also been described in PR-positive T-47D breast cancer cells in which PRA can be induced to result in PRA predominance. Cell adhesion of T-47D cells was decreased upon progestin treatment and reduced even further with PRA predominance [11]. Another enriched biological process identified in this study for downregulated genes was transmembrane transport, and the cell membrane itself was enriched amongst the cellular compartments. Similarly, Richer et al. found an extensive number of genes involved in membrane-initiated events that were regulated by PR isoforms in response to progesterone, thus stressing the membrane as an important target of progesterone action [33]. Also, more recently, SLC- mediated transmembrane transport was found amongst enriched pathways in human breast PRA high tumors as compared to PRB high tumors [10].

Then, we used GSEA to identify pathways and unifying themes. The GSEA method focuses on gene sets rather than a handful of high scoring genes at the top and bottom (which can suffer from arbitrary cutoff regarding fold-change or significance) giving more reproducible and easy to interpret results [20]. When necessary, we plotted enrichment maps of gene sets to aid interpretation [21]. Interestingly, we found a positive correlation of PRA transgenics with gene sets comprising amplicons previously identified in human breast cancer (Fig. 3a). One example is amplicon 16p13, which was previously correlated with luminal breast cancer subtype and comprises effector proteins such as proteases [28]. For genes downregulated in PRA transgenics, we identified three clusters based on the enrichment map (Fig. 3b). Not surprisingly, one cluster (ii) included gene sets related to mammary gland morphological changes or breast cancer subtypes. For example, a set comprising genes downregulated in ductal carcinoma vs normal ductal breast cells identified by laser microdissection and microarray analysis [34] and another set of genes that were downregulated in HMLE cells (immortalized nontransformed mammary epithelial) cells after loss of function of E-cadherin (CDH1) achieved by RNAi knockdown or by expression of a dominant-negative form [35]. Of note, mammary glands of PRA transgenics exhibited diminished E-cadherin in a disorganized pattern [13]. Another region in the map (ii) clustered gene sets related to estradiol or tamoxifen response like one set of genes downregulated in breast cancer SUM44/LCCTam cells resistant to 4-hydroxytamoxifen relative to the parental sensitive cells [36]. Importantly, in PR-positive breast cancer patients who received local therapy followed by adjuvant tamoxifen, high PRA:PRB ratios predicted shorter disease-free survival, indicating resistance to tamoxifen [7]. Our third (iii) identified cluster included gene sets comprising targets of polycomb complexes. In particular, Polycomb Repression Complex 2 (PRC) targets that possess H3K27me3 mark in their promoters and are bound by SUZ12 and EED Polycomb proteins [37]. This suggests that PRC2 may be an upstream regulator accounting for the high number of downregulated genes in PRA transgenics. Interestingly, H3K27me3 mark has been positively associated with the LumA subtype compared to all other subtypes in a cohort of breast cancer patients [38].

We then performed GSEA analysis using the Hallmark collection (Table 2). We found positive enrichments mainly in metabolic pathways for the PRA transgenics phenotype. Changes in metabolism during tumorigenesis are well known. Our analysis suggests metabolic plasticity, as oxidative phosphorylation in addition to glycolysis were significantly enriched, and this adaptability may be important as tumorigenesis progresses [39]. Pathways negatively correlating with PRA transgenics phenotype pointed to an anti-inflammatory effect. PR action has been linked previously with inhibition of inflammatory response in breast cancer cells [40] and myometrial cells [41]. However, the uterine phenotype of PRA transgenics included endometritis and pelvic inflammatory disease (together with hyperplasia) [42], therefore effects on inflammation may be context-dependent in PRA transgenics. Recently, Cai et al. analyzed gene expression from four distinct stages of mammary tumor progression using the MMTV-PyMT mouse model and found similar enriched hallmark pathways amongst up and downregulated genes in the hyperplasia stage [43]. For example, apical junction, epithelial to mesenchymal transition, UV response, KRAS signaling up, IL2 STAT5 signaling and hypoxia (all also enriched in the present study) were enriched in the down-regulated DEGs at normal to premalignant (hyperplasia) transition in the MMTV-PyMT model. Importantly, most DEGs identified in the same study in the late carcinoma stage first appeared in the much earlier hyperplasia stage, consistently with previous human cancer studies [29, 30]. This prompted us to compare the hallmark pathways identified for PRA transgenics to those obtained using the same methodology in a much larger data set of human breast cancer samples [22, 23]. Interestingly, we found significant overlapping between pathways exclusively with luminal breast cancer subtypes (Table 4). Rojas et al. have recently classified human breast tumors according to their PRA:PRB ratio (high versus low) by western blot detection and predicted according to the PAM50 gene set that PRB-High and PRA-High tumors were either luminal B or A phenotypes, respectively [10]. It would be interesting to determine by the same method the intrinsic subtype based on PRA transgenics gene expression profile, as at least by our approach overlapping with luminal B subtype is predicted according to pathway analysis. Finally, we explored the potential prognostic value of common candidate genes from the most significantly enriched pathways and found an association with worse relapse free survival and high PDHB (upregulated in PRA transgenics, Fig. 4) or low LAMB3 (downregulated in PRA transgenics, Fig. 5) for luminal breast cancer subtypes. To the best of our knowledge, this is the first report of the potential prognostic value of this particular laminin subunit and PDHB in breast cancer.

## Conclusion

Further characterization of these and other genes identified in the present study would greatly increase our understanding of the early tumorigenic events associated with high PRA:PRB ratio and the underlying biological mechanisms and may provide new prognostic markers for breast cancer.

## Additional files

Additional file 1: **Table S1.** Provides a complete list of the genes identified by GSEA analysis contributing to the upregulated oxidative phosphorylation pathway in both PRA transgenics and LumB breast cancer subtype. **Figure S1.** Provides TIMM9 expression, from the oxidative phosphorylation pathway, in samples from the METABRIC study, classified by subtype and relapse-free survival curves according to TIMM9 expression for the luminal breast cancer subtypes. **Table S2.** Provides a complete list of the genes identified by GSEA analysis contributing to the downregulated TNFA signaling via NFKB pathway in PRA transgenics, LumA and LumB breast cancer subtypes. **Figure S2.** Provides expression of genes, other than LAMB3, from the TNFA signaling through NFKB pathway in samples from the METABRIC study, classified by subtype. **Figure S3.** Provides relapse-free survival according to expression of the genes presented in Figure S2. for the luminal breast cancer subtypes. (PDF 5906 kb)

## Abbreviations

BiNGO: Biological network gene ontology
DEG: differentially expressed genes
ER: estrogen receptor
FC: fold changes
GO: Gene ontology
GSEA: Gene set enrichment analysis
LAMB3: laminin subunit beta 3
LumA: Luminal A
LumB: Luminal B
PDHB: Pyruvate dehydrogenase E1 component subunit beta, mitochondrial
PR: Progesterone receptor
PRA transgenics: Transgenic mice carrying an additional A form of progesterone receptor
PRA: Progesterone receptor isoform A
