# Supplementary material for: Gene expression profile and cancer-associated pathways linked to progesterone receptor isoform a (PRA) predominance in transgenic mouse mammary glands
Source: BMC Cancer. 2018 Jun 25;18:682. doi: 10.1186/s12885-018-4550-z (PMC6019805; doi:10.1186/s12885-018-4550-z)
Supplement: Supplementary file 1 — Table S1. Provides a complete list of the genes identified by GSEA analysis contributing to the upregulated oxidative phosphorylation pathway in both PRA transgenics and LumB breast cancer subtype. Figure S1. Provides TIMM9 expression, from the oxidative phosphorylation pathway, in samples from the METABRIC study, classified by subtype and relapse-free survival curves according to TIMM9 expression for the luminal breast cancer subtypes. Table S2. Provides a complete list of the genes identified by GSEA analysis contributing to the downregulated TNFA signaling via NFKB pathway in PRA transgenics, LumA and LumB breast cancer subtypes. Figure S2. Provides expression of genes, other than LAMB3, from the TNFA signaling through NFKB pathway in samples from the METABRIC study, classified by subtype. Figure S3. Provides relapse-free survival according to expression of the genes presented in Figure S2. for the luminal breast cancer subtypes. (PDF 5906 kb) [file 12885_2018_4550_MOESM1_ESM.pdf]

Table S1. Genes identified by GSEA analysis contributing to the upregulated oxidative phosphorylation pathway in both PRA transgenics and LumB breast cancer subtype. Genes that were also in our original list and are highlighted.

**Oxidative phosphorylation pathway genes (GSEA analysis)**
ATP5B | ATP5G3 | ATP5J | ATP6AP1 | ATP6V0C | ATP6V1C1 | DLAT | GRPEL1 | HSD17B10 | ISCA1 LDHA | LRPPRC | MDH1 | MRPL15 | MRPL35 | MRPS11 | MTRR | NDUFB2 | NDUFS2 | NDUFS8 | NDUFV2 | NNT | OPA1 | **PDHB** | PDHX | SDHB | **TIMM9** | UQCRB | UQCRH

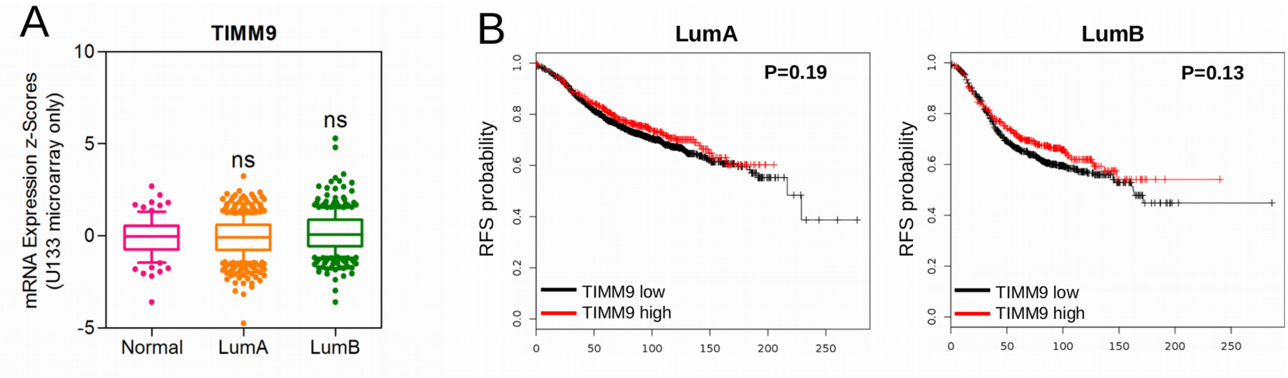

Figure S1. A) TIMM9 expression, from the oxidative phosphorylation pathway, in samples from the METABRIC study, classified by subtype. ns=not significant, One-way ANOVA Tukey's multiple comparison test. B) Relapse-free survival curves according to TIMM9 expression for the luminal breast cancer subtypes.

Table S2. Genes identified by GSEA analysis contributing to the downregulated TNFA signaling via NFKB pathway in PRA transgenics, LumA and LumB breast cancer subtypes (with few exceptions). Genes that were also in our original list and are highlighted.

**TNFA signaling via NFKB pathway genes (GSEA analysis) identified in PRA transgenics, LumA and Lumb breast cancer subtypes**
ATF3 | B4GALT5 | **BMP2** | BTG1 | **CCL2** | CCL5 | CD69 | CEBPB | CEBPD | CXCL1 | CXCL2 | CYR61 | DUSP1 | **EGR1** | EGR2 | **EGR3** | EHD1 | F3 | FOS | **FOSB** | GEM | HBEGF | **IER2** | IER5 | **IL7R** | IRS2 | JAG1 | JUN | **JUNB** | KLF10 | **KLF2** | KLF4 | **LAMB3** | MAP3K8 | NFKBIA | **NR4A1** | **PDE4B** | PPAP2B | PPP1R15A | PTGER4 | PTGS2 | PTPRE | RCAN1 | SERPINE1 | SIK1 | SLC2A3 | TGIF1 | TNFAIP3 | TNFAIP8 | TRAF1 | **ZFP36**

**Genes identified in PRA trasgenics and LumA breast cancer subtype exclusively**
DUSP2 | **KYNU** | SMAD3

**Genes identified in PRA trasgenics and LumB breast cancer subtype exclusively**
BTG2 | **CD44** | GADD45B | GFPT2 | HES1 | KDM6B | **MSC** | **PMEPA1**

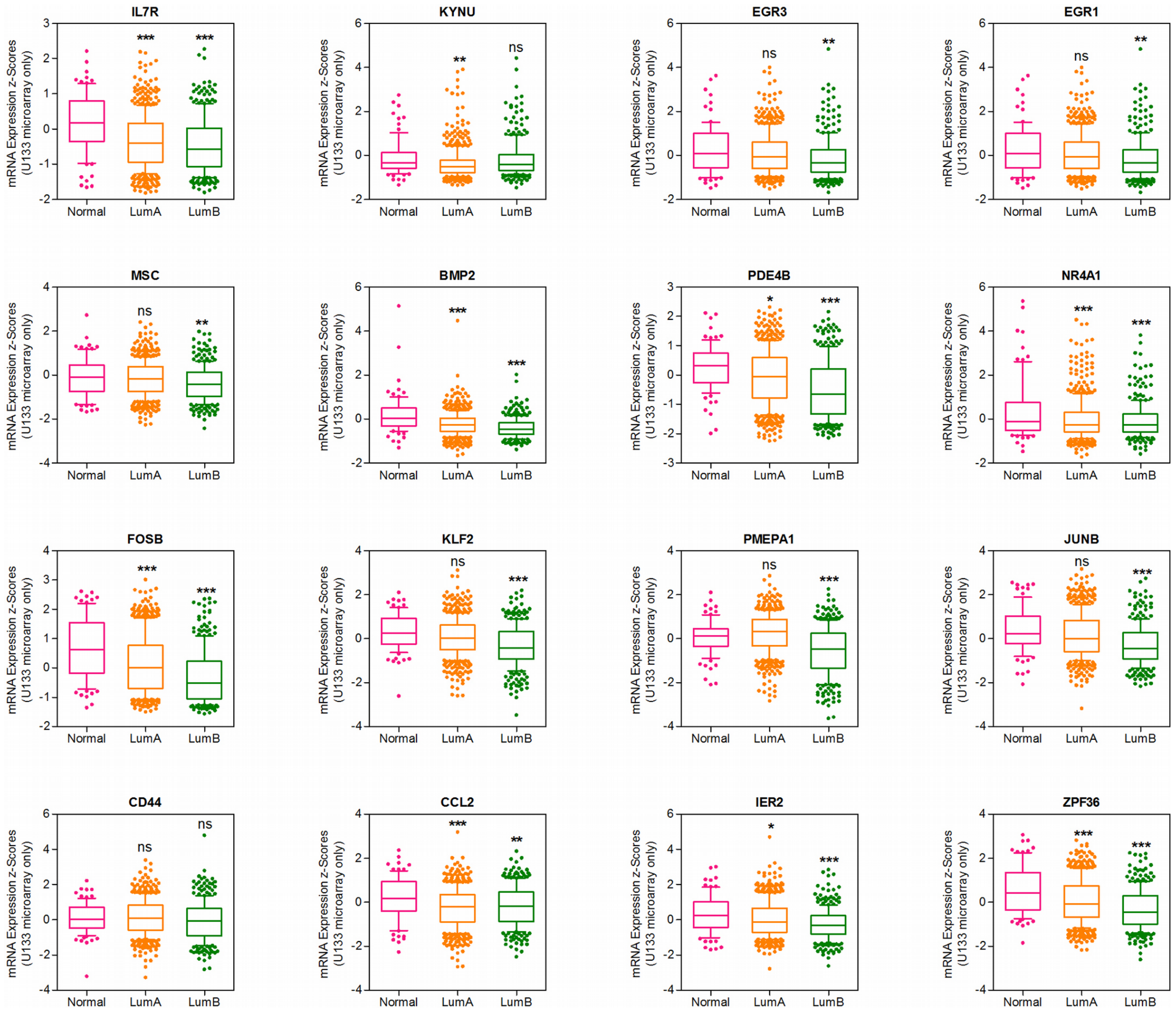

Figure S2. Expression of genes from the TNFA signaling through NFKB pathway (Table 4) in samples from the METABRIC study, classified by subtype. ***P<0.001, **P<0.01, *P<0.05, ns=not significant, One-way ANOVA Tukey's multiple comparison test.

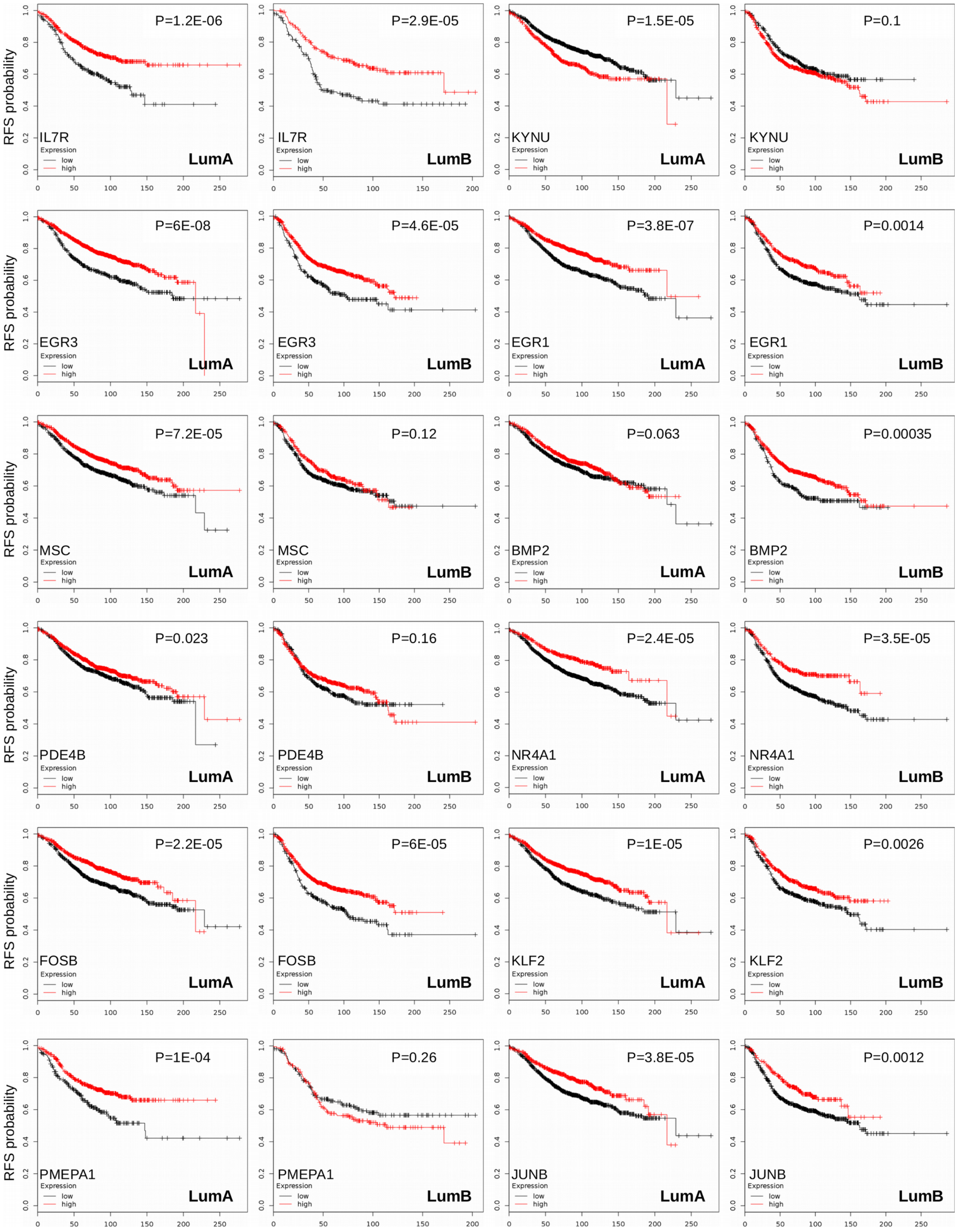

Figure S3. Relapse-free survival curves according to expression of the genes presented in Figure S2 for the luminal breast cancer subtypes.

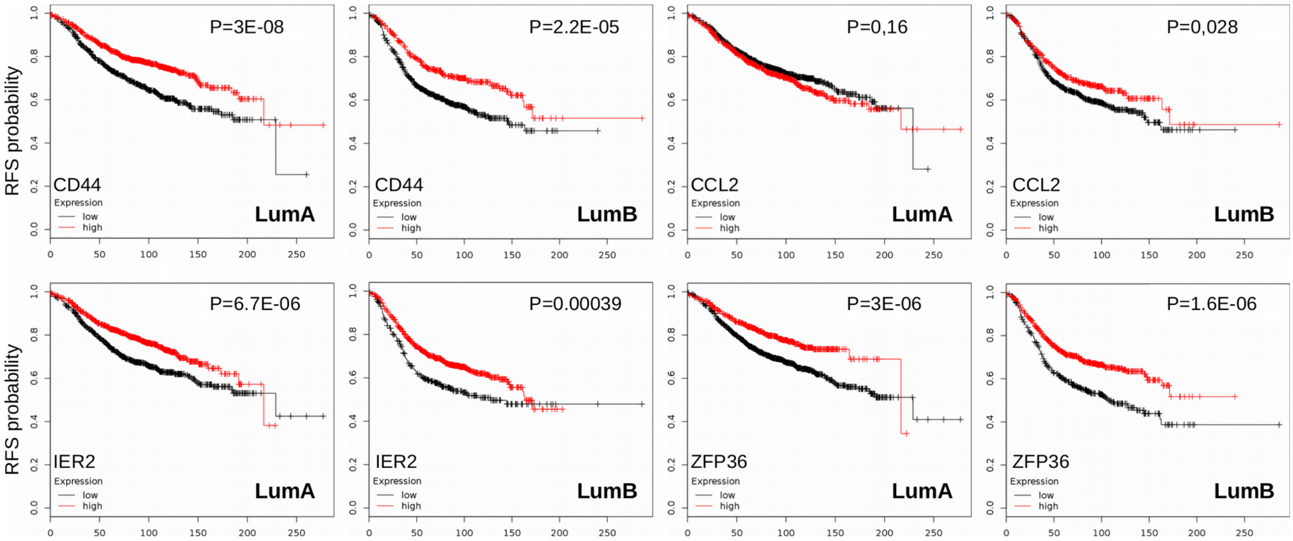

Figure S3 continued.
